# Assembly-driven activation of the AIM2 foreign-dsDNA sensor provides a polymerization template for downstream ASC

**DOI:** 10.1038/ncomms8827

**Published:** 2015-07-22

**Authors:** Seamus R. Morrone, Mariusz Matyszewski, Xiong Yu, Michael Delannoy, Edward H. Egelman, Jungsan Sohn

**Affiliations:** 1 Department of Biophysics and Biophysical Chemistry, Johns Hopkins University School of Medicine Baltimore, Maryland 21205, USA; 2 Department of Biochemistry and Molecular Genetics, University of Virginia School of Medicine Charlottesville, Virginia 22908, USA; 3 Microscope Core Facilities, Johns Hopkins University School of Medicine, Baltimore, Maryland 21205, USA

## Abstract

AIM2 recognizes foreign dsDNA and assembles into the inflammasome, a filamentous supramolecular signalling platform required to launch innate immune responses. We show here that the pyrin domain of AIM2 (AIM2^PYD^) drives both filament formation and dsDNA binding. In addition, the dsDNA-binding domain of AIM2 also oligomerizes and assists in filament formation. The ability to oligomerize is critical for binding dsDNA, and in turn permits the size of dsDNA to regulate the assembly of the AIM2 polymers. The AIM2^PYD^ oligomers define the filamentous structure, and the helical symmetry of the AIM2^PYD^ filament is consistent with the filament assembled by the PYD of the downstream adaptor ASC. Our results suggest that the role of AIM2^PYD^ is not autoinhibitory, but generating a structural template by coupling ligand binding and oligomerization is a key signal transduction mechanism in the AIM2 inflammasome.

## Abstract (web summary)

The AIM2 inflammasome complex is essential for defence against a number of human pathogens but how it assembles upon recognition of foreign DNA remains incompletely understood. Here Morrone *et al.* suggest the AIM2 pyrin domain acts in both DNA binding and filament assembly to generate a structural template for complex assembly.

## Graphical abstract

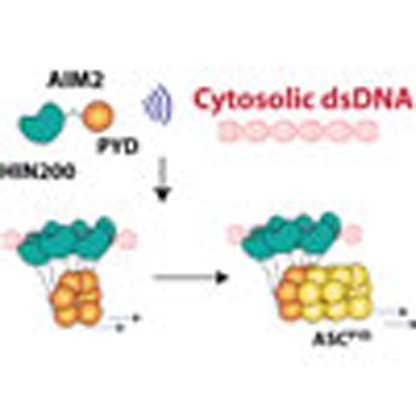

In the innate immune system of mammals, supramolecular signalling platforms are directly assembled on intracellular foreign double-stranded (ds) DNA and RNA arising from invading pathogens (see refs 1, 2, 3, 4, 5, 6 for review). Unlike conventional enzymatic turnovers, these supra-structures then induce the sequential polymerization of downstream effectors to propagate upstream signals^7^,^8^,^9^,^10^. Though essential for defence against a number of pathogens such as *Francisella tularensis* and herpes simplex viruses^11^,^12^,^13^, dysregulated foreign-nucleic acid-sensing pathways are associated with several autoimmune disorders including systemic lupus erythematosus and Sjögren's syndrome^14^,^15^,^16^. How the assembly of these large, complex structures is initiated on appropriate nucleic acids, and how the upstream ligand·receptor assemblies promote the sequential oligomerization of specific downstream effectors are two major unresolved mechanistic questions in understanding the foreign-nucleic acid-sensing pathways^5^,^6^. In this report, we set out to answer these fundamental questions in the assembly of the foreign-dsDNA-sensing filamentous superstructures by absent-in-melanoma-2 (AIM2).

AIM2 is a prototypical member of the AIM2-like receptor (ALR) family, which also includes other major foreign-dsDNA sensors such as interferon-inducible protein 16 (IFI16) (refs 17, 18, 19, 20, 21). ALRs directly assemble filamentous signalling platforms termed the inflammasomes on foreign dsDNA^7^,^18^,^19^,^20^,^21^,^22^,^23^,^24^,^25^. For instance, AIM2 oligomerizes on cytosolic dsDNA and nucleates the polymerization of the ASC (apoptosis-associated speck-forming protein containing CARD (caspase-recruiting domain)) adaptor filament, which then nucleates the polymerization of the procaspase-1 filament; this final polymerization step activates caspase-1 via auto-proteolysis, triggering inflammatory responses including cytokine maturation and pyroptosis (Fig. 1a)^7^,^8^,^18^,^19^,^20^,^21^.

Many proteins build nucleoprotein filaments, frequently by classic RecA-like mechanisms^9^,^10^,^26^,^27^. Indeed, retinoic acid-inducible gene-I (Rig-I) and myeloid-differentiation-antigen-5 (MDA5) directly bind foreign dsRNA and assemble into filamentous signalling platforms using their RecA-like helicase domains^9^,^10^,^28^,^29^,^30^. However, ALRs are not RecA-like proteins, but consist of one oligomerization domain named pyrin domain (PYD) and one or two nonspecific dsDNA-binding HIN200 (hematopoietic interferon-inducible nuclear protein with 200 amino acids) domains (Fig. 1a)^31^. Indeed, in contrast to Rig-I or MDA5 (refs 9, 10, 28, 29, 30), our recent study of IFI16 suggests that nucleic acid binding and polymerization are allosterically coupled in ALRs^23^. For instance, the two HIN200 domains of IFI16 in isolation not only lack any significant oligomerization activity but also fail to form stable dsDNA-bound complexes under physiologically relevant reaction conditions^23^. However, such a transient interaction between the HIN200 domains and dsDNA larger than 60 base pairs (bp) permits at least four IFI16 molecules to cluster, allowing the non-dsDNA-binding PYD of IFI16 (IFI16^PYD^) to initiate the assembly of the filament in a switch-like manner^23^.

There are two major unresolved questions regarding AIM2 inflammasome assembly. First, how AIM2 initiates the assembly is controversial. In contrast to the essential positive function of IFI16^PYD^ in dsDNA binding and oligomerization^22^,^23^, it was reported that the PYD of AIM2 (AIM2^PYD^) plays an autoinhibitory function by blocking the dsDNA-binding surface of HIN200 domain (AIM2^Hin^) (refs 32, 33). Although it was recently reported that isolated AIM2^PYD^ auto-assembles into filaments^7^,^34^, whether the filamentation activity has any role in dsDNA binding remains unknown. Second, although it has been established that AIM2^PYD^ directly induces the polymerization of ASC^PYD^ (ref. 7), how the recognition occurs at the structural level remains speculative. For instance, many inflammatory signalling proteins contain PYDs and several highly conserved side chains that mediate multimeric PYD–PYD interactions have been identified^7^,^34^,^35^; however, only a subset of PYDs is known to interact with ASC^36^,^37^. A recent study on foreign-dsRNA-sensing pathways provides a potential mechanism, as Hur and colleagues discovered that congruent multimeric architectures underpin the sequential oligomerization in the Rig-I signalling pathway^9^. For instance, Rig-I recruits the CARDs of mitochondrial antiviral-signalling protein (MAVS^CARD^) into the helical ‘oligomerization trajectory' of its CARD tetramers (Rig-I^CARD^), consequently providing a structural template for the polymerization of the MAVS^CARD^ filament^9^. Wu and colleagues have determined the architecture of the filament assembled by the PYD of ASC (ASC^PYD^) (ref. 7). However, because the architecture of AIM2^PYD^ filament is unknown, it remains to be seen whether the AIM2–ASC axis operates in a similar manner.

Here we find that AIM2 is not autoinhibited, but the size of dsDNA can act as a ‘molecular ruler' to regulate the oligomerization of AIM2. We also find that the helical symmetry of the AIM2^PYD^ filament is consistent with that of ASC^PYD^, suggesting that AIM2^PYD^ filaments provide a template for assembling AIM2 filaments.

## Results

### N-terminal MBP masks the oligomerization activity of AIM2

No biochemical studies reported to date employ full-length AIM2 (AIM2^FL^) without additional protein tags. Indeed, the cornerstone of the autoinhibitory model proposed by Xiao and colleagues is an observation where N-terminal maltose-binding protein (MBP)-tagged full-length AIM2 (AIM2^FL^) binds dsDNA more weakly than isolated AIM2^Hin^ (ref. 32). However, it was shown that N-terminal MBP masks the intrinsic oligomerization activity of PYDs by blocking a key interaction interface^7^,^33^,^34^. Thus, to re-examine the initiation of the AIM2 inflammasome assembly, we first generated recombinant AIM2^FL^ and the isolated AIM2^Hin^ in which N-terminal MBP tags are either left in place or removed by the tobacco etch virus (TEV) protease. The resulting AIM2 constructs all purify as monomers in size-exclusion chromatography (Supplementary Fig. 1a,b). We compared the binding affinity of MBP-tagged and -untagged AIM2 variants by monitoring changes in fluorescence anisotropy of fluorescein amidite (FAM)-labelled 72-bp dsDNA (that is, FAM-dsDNA72).

**Figure 1:**
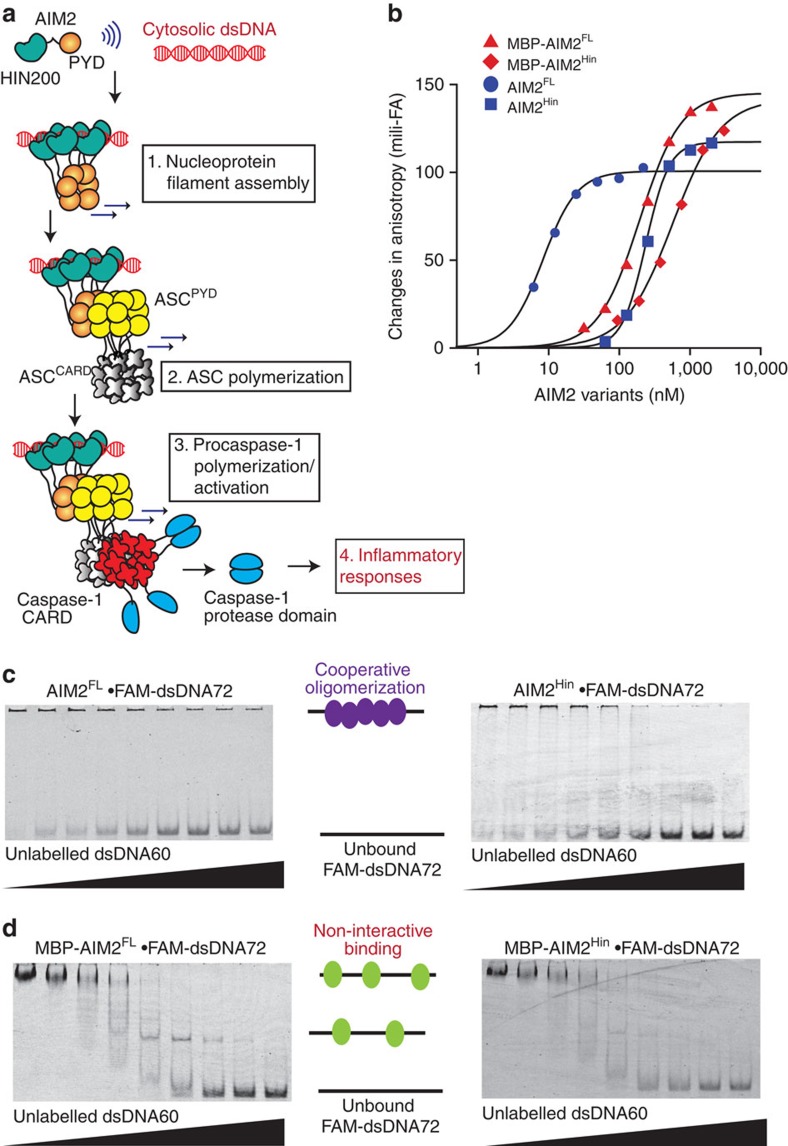
Oligomerization is integral to dsDNA binding by AIM2. (**a**) A model for the assembly of the AIM2 inflammasome on foreign dsDNA. AIM2 is comprised of one PYD that oligomerizes and one dsDNA-binding HIN200 domain, flanked by an unstructured 50 amino acid-linker region. ASC is a bipartite protein containing one PYD and one CARD. Procaspase-1 contains one CARD followed by the protease domain. Only a few protease domains are shown for simplicity. (**b**) Binding of AIM2 variants to FAM-dsDNA72 (2.5 nM) was monitored by changes in fluorescence anisotropy. The lines are fits to a Hill form of binding isotherm. The apparent binding constants (K_D_) are determined by the Hill equation (bound=1/(1+(K_D_/(AIM2))^Hill constant^)) and the values are listed in Supplementary Table 1. All presented experiments were performed at least three times. (**c**,**d**) Competition EMSAs in which increasing concentrations of dsDNA60 (190, 95, 45, 23, 12, 6, 3 and 1.5 μg ml^−1^) were added to AIM2 (variants) FAM-dsDNA72 (200 nM and 0.2 μg ml^−1^, respectively).

MBP-AIM2^FL^ bound FAM-dsDNA72 nearly twofold more tightly than MBP-AIM2^Hin^ (Fig. 1b; Supplementary Table 1). Without MBP, AIM2^FL^ bound dsDNA72 at least 20-fold more tightly than isolated AIM2^Hin^. Moreover, both tag-less AIM2 variants bound dsDNA72 significantly more tightly than their MBP-tagged counterparts (Fig. 1b). Unlike the other AIM2 variants, the apparent binding constant of AIM2^FL^ changed with FAM-dsDNA72 concentrations in our assays (compare Fig. 1b with Supplementary Fig. 1c), indicating that it binds even more tightly than the detection limit of our instrument (∼0.5 nM). These data suggest that N-terminal MBP interferes with dsDNA binding of both AIM2^FL^ and isolated AIM2^Hin^, and that AIM2^PYD^ plays a major positive role in dsDNA binding by AIM2.

Competition electrophoretic mobility shift assays with dsDNA fragments containing multiple AIM2^Hin^-binding sites can test whether the initiation of the AIM2 inflammasome assembly on dsDNA proceeds via a two-state oligomerization binding mode^23^,^28^as opposed to the previously proposed non-interactive binding mechanism^32^,^33^: no partially occupied intermediate dsDNA species are observed when a competitor is included^23^,^28^. Here as observed for full-length IFI16 (ref. 23), both AIM2^FL^ and isolated AIM2^Hin^ showed an all-or-none transition; however, the MBP-tagged variants showed intermediates, further supporting a negative effect of MBP tags in the oligomerization-driven dsDNA binding of AIM2 (Fig. 1c versus d).

### dsDNA binding and oligomerization is integrated

**Figure 2:**
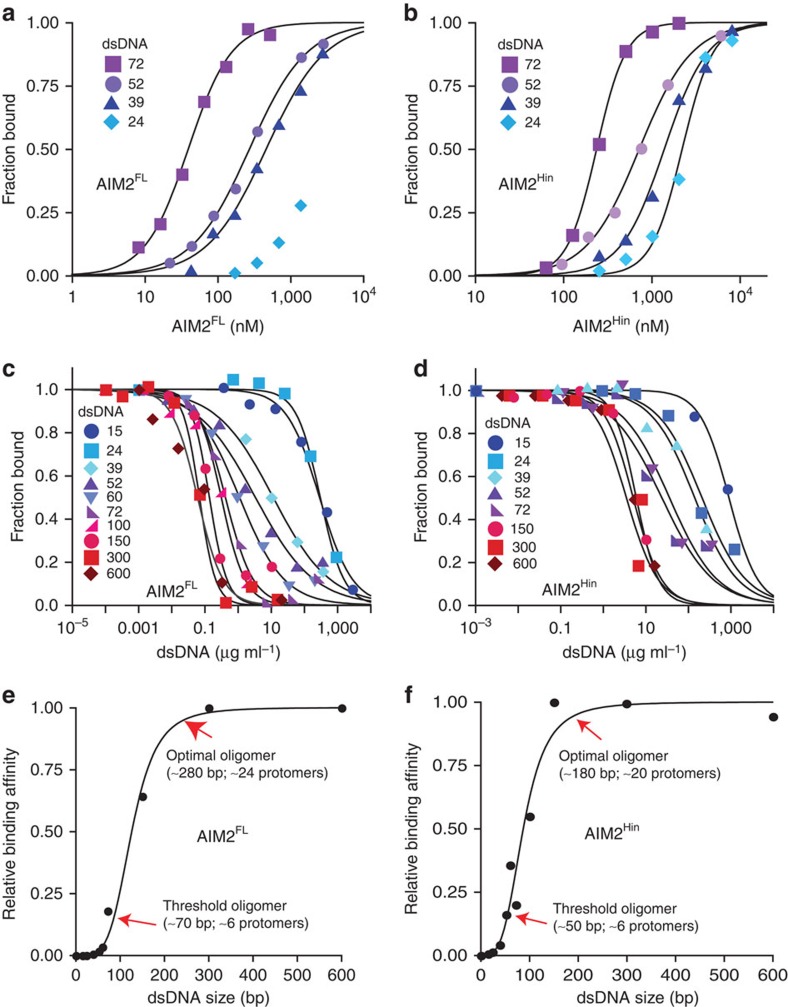
AIM2^FL^ and isolated AIM2^Hin^ bind dsDNA in a length-dependent manner. (**a**) Binding of AIM2^FL^ and (**b**) isolated AIM2^Hin^ to each FAM-labelled dsDNA (1.5 nM) was determined by fluorescence anisotropy. The determined K_D_ values are listed in Supplementary Tables 2–4. (**c**) Competition binding assays using FAM-dsVACV72 (1.5 nM, 0.06 μg ml^−1^) and AIM2^FL^ (70 nM) at 400 mM KCl against various dsDNA fragments; the lines are fits to a competition binding equation: 1/(1+((DNAcompetitor)/IC_50_)^Hill constant^). The determined IC_50_ values are listed in Supplementary Tables 5 and 7. (**d**) Competition binding assays using FAM-dsVACV72 (5 nM, 0.2 μg ml^−1^) and AIM2^Hin^ (250 nM) at 160 mM KCl against various DNA fragments. The determined values are listed in Supplementary Table 6. The plots of the binding efficiency versus the length of dsDNA for AIM2FL (**e**) and AIM2Hin (**f**). The binding efficiency was determined by normalizing the mean IC_50_ of each fragment to that of dsDNA600, and the data were fit to the Hill equation (the Hill constant for (**e**) is 4.2±0.2 and (**f**) is 3.7±0.3; ±indicates s.d., *n*≥3). The ‘threshold' oligomer is defined as the size of dsDNA (AIM2 cluster) required to exit the apparent lag phase, and the ‘optimal' oligomer is the size of dsDNA (AIM2 cluster) required to reach the inflection point.

We then tested whether the dsDNA-binding affinity of AIM2 changes with the size of dsDNA, because such a behaviour is expected when ligand binding is dependent on oligomerization^13^,^23^. For instance, the apparent binding affinity would increase nonlinearly up to the ‘optimal' oligomer dictated by nucleic acid sizes^23^,^28^. To characterize the DNA-binding property of AIM2^FL^ within the detection limit of our instruments, we used the reported salt concentration-dependent binding of AIM2^Hin^ (ref. 32) and performed binding assays with various dsDNA sizes at 400 mM KCl (Fig. 2). Even in this high salt condition, AIM2^FL^ robustly bound FAM-dsDNA72 (Fig. 2a; the binding constant did not change with FAM-dsDNA72 concentrations, see Supplementary Fig. 1d). Importantly, AIM2^FL^ bound the larger dsDNA more tightly (Fig. 2a), suggesting that oligomerization is integral to dsDNA binding. However, MBP-AIM2^FL^, MBP-AIM2^Hin^ and AIM2^Hin^ all showed no detectable binding in this high salt condition (Supplementary Fig. 1e), again supporting the positive role of AIM2^PYD^ in dsDNA binding. In 160-mM KCl, isolated AIM2^Hin^ also bound the larger dsDNA more tightly (Fig. 2b). Finally, both AIM2^FL^ and isolated AIM2^Hin^ bound the footprint-size dsDNA (10 bp) with minimal affinity (Supplementary Fig. 1f), suggesting that oligomerization is important for high affinity binding of both AIM2 variants (the dsDNA-binding footprint of AIM2^Hin^ is ∼9 bp^32^ and that of AIM2^FL^ is ∼12 bp, Supplementary Fig. 1g).

Next, we performed competition binding assays to further investigate the relationship between dsDNA size and binding efficiency (we used dsDNA mass concentrations to normalize the number of available binding sites in each competitor; Supplementary Tables 5–7). Consistent with the direct binding data, both AIM2^FL^ and isolated AIM2^Hin^ bound larger dsDNA fragments significantly more tightly (Fig. 2c,d). We used 160 mM KCl to allow isolated AIM2^Hin^ to bind dsDNA; the dsDNA size-dependent binding was apparent for AIM2^FL^ at both 400 and 160 mM KCl, confirming that buffer salt concentrations do not alter the overall mechanism (see Supplementary Fig. 1h,i for 160 mM). The plots of half-maximal inhibition (IC_50_) versus dsDNA size revealed a sigmoidal relationship where the difference in binding affinity between the near footprint-size dsDNA (dsDNA15) and dsDNA fragments larger than 300 bp can be as much as 4,000-fold for AIM2^FL^ and 150-fold for AIM2^Hin^, respectively (Fig. 2e,f). We also assessed the cooperativity between the binding affinity and dsDNA size by fitting the data using the Hill equation (Fig. 2e,f). The fitted Hill constant near four in Fig. 2e suggests that the binding efficiency of AIM2^FL^ would improve 10,000-fold when the size of dsDNA is increased by only 10-fold. Furthermore, about 70-bp dsDNA was required to exit the lag phase (‘threshold') and about 250- to 300-bp dsDNA fragments were required to achieve the ‘optimal' efficiency for generating AIM2^FL^·dsDNA complexes (Fig. 2e). These dsDNA sizes in turn indicate that about 6 AIM2^FL^ molecules are required to assemble a ‘threshold' oligomer and about 24 AIM2^FL^ molecules will generate an ‘optimal' oligomer (Fig. 2e). The observed dsDNA size-dependent binding also correlates with a previous *in vivo* observation in which the interleukin-1β secretion activity increased cooperatively between 10 and 80 bp transfected dsDNA^32^. Overall, the ‘digitized' nucleoprotein complex-forming activity of AIM2 suggests that dsDNA can act as a ‘molecular ruler' to control the assembly of the inflammasome in a switch-like mechanism.

### Mutagenesis studies support the positive role of AIM2^PYD^

**Figure 3:**
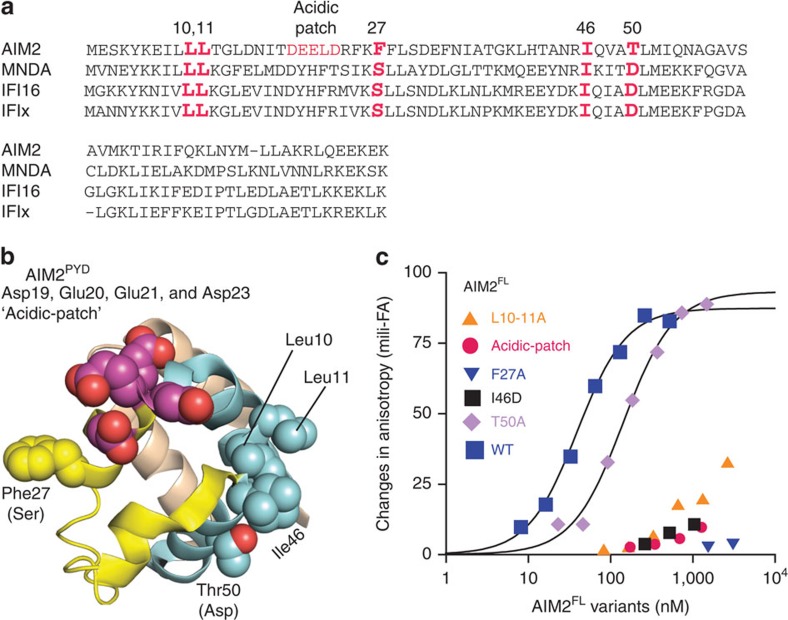
Mutagenesis studies to test the role of AIM2^PYD^ in dsDNA binding. (**a**) The sequence alignment of ALRs. The mutated side chains are indicated in red. (**b**) The crystal structure of AIM2^PYD^ (PDB ID: 3VD8). The mutated side chains are shown as spheres. The amino acids indicated in the parentheses are the equivalent IFI16 residues. (**c**) Binding of FAM-dsDNA72 (1.5 nM) by various AIM2^FL^ PYD mutants were tested at 400 mM KCl. The determined K_D_ values are listed in Supplementary Table 2.

Previously, we found that several highly conserved surface side chains of IFI16 mediate its oligomerization-driven dsDNA-binding mechanism^23^ (Fig. 3a). For AIM2, several equivalently positioned surface side chains mediate the auto-oligomerization of isolated AIM2^PYD^ *in vivo*^34^ and interaction with ASC^PYD^ *in vitro* 7 (for example, Leu10, Leu11 and Phe27 in Fig. 3b). However, whether these side chains have any role in dsDNA binding remains unknown. To further test the positive role of AIM2^PYD^ in dsDNA binding, we then generated a panel of surface mutants based on our study of IFI16 (ref. 23) and sequence conservation (Fig. 3a,b; none of these side chains are implicated in the alleged autoinhibition of AIM2 (refs 32, 33), see also below). Almost all of these ^PYD^ mutants significantly disrupted the dsDNA-binding activity of AIM2^FL^ (Fig. 3c), supporting that oligomerization of PYD plays a major positive function in dsDNA binding.

Xiao and colleagues proposed that a unique acidic surface of AIM2^PYD^ not present in other related ALR docks to the basic dsDNA-binding surface of AIM2^Hin^, thus stabilizing the autoinhibited conformation in the absence of dsDNA33 (Fig. 3a,b; designated ‘acidic patch'; Asp19, Glu20, Glu21 and Asp23). Such an autoinhibitory model entails that neutralizing the acidic patch would allow AIM2^FL^ to bind dsDNA more tightly. However, D19A-E20A-E21A-D23A-AIM2^FL^ failed to bind FAM-dsDNA72 (Fig. 3c). This observation disagrees with the inhibitory role of the acidic patch, but is again consistent with the idea that AIM2^PYD^ plays a positive role in dsDNA binding.

### The oligomerization of AIM2^Hin^ is shared with murine p202

**Figure 4:**
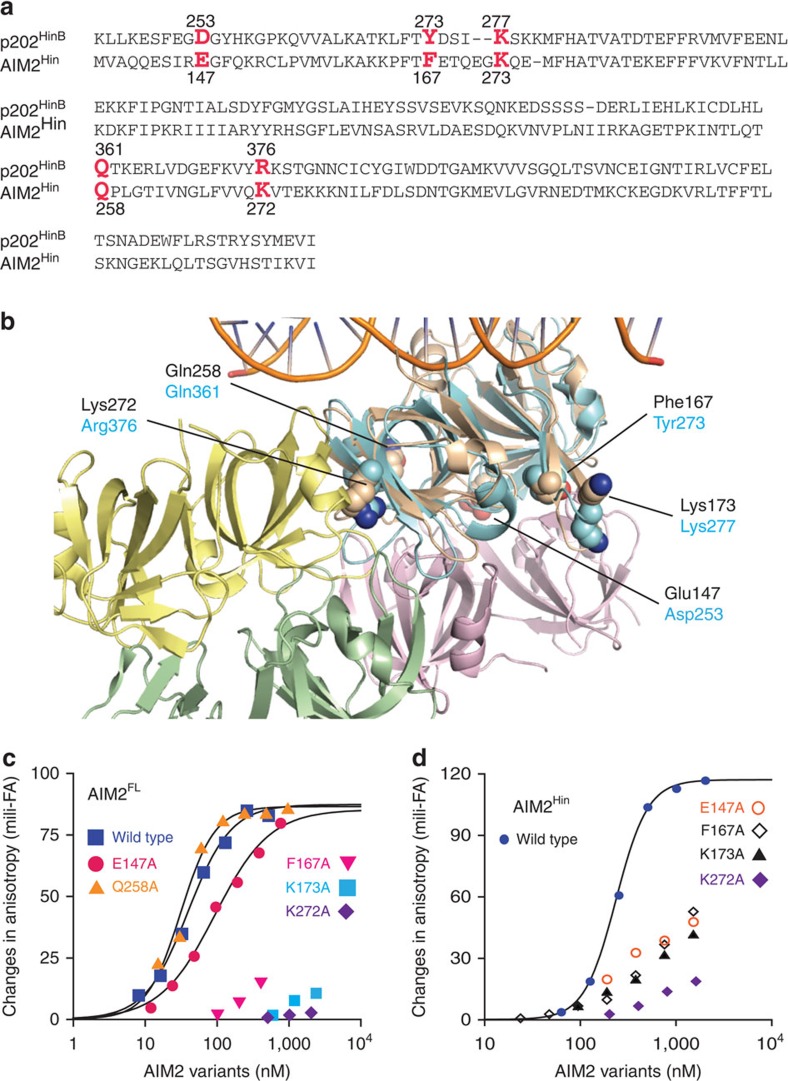
Mutagenesis studies to test the evolutionarily conserved oligomerization activity AIM2^Hin^ in dsDNA binding. (**a**) The sequence alignment of AIM2^Hin^ and p202^HinB^. The mutated side chains are indicated in red. (**b**) The crystal structure of dsDNA-bound AIM2^Hin^ (coloured in ‘wheat;' PDB ID: 3RN2) aligned to the p202^HinB^ tetramer structure (PDB ID: 4L5T); root mean squared deviation of alignment is 1.5 Å. The p202^HinB^ protomers are coloured in green, yellow, pink and cyan, respectively. The mutated side chains are shown as spheres. AIM2 side chains are labelled in black and those of p202 are indicated in cyan. (**c**) Binding of FAM-dsDNA72 (1.5 nM) by various AIM2^FL^ HIN200 mutants were tested at 400 mM KCl. The determined K_D_ values are listed in Supplementary Table 2. (**d**) Binding of various AIM2^Hin^ mutants were tested on FAM-dsDNA72 (5 nM) at 160 mM KCl. The determined K_D_ values are listed in Supplementary Table 2.

In contrast to the dsDNA-binding HIN200 domains of related IFI16 (ref. 23), our dsDNA-binding studies suggest that AIM2^Hin^ alone oligomerizes on dsDNA (Figs 1c and 2b,d,f). On the other hand, a murine ALR named p202 inhibits the activity of AIM2 by binding to AIM2^Hin^ with its tetrameric second HIN200 domain (p202^HinB^); p202^HinB^ does not bind dsDNA^38^. Several p202^HinB^ side chains implicated in tetramerization^38^ are conserved in AIM2^Hin^ (Fig. 4a,b). To test whether AIM2^Hin^ uses similarly positioned side chains as p202^HinB^ to cluster on dsDNA, we mutated the indicated side chains in Fig. 4a,b on both AIM2^FL^ and isolated AIM2^Hin^. We found mutations distal to the dsDNA-binding surface significantly decreased dsDNA binding by both AIM2^FL^ and AIM2^Hin^ (Fig. 4c,d). These results not only support the idea that the oligomerization of AIM2^Hin^ is important for dsDNA binding but also suggest that the oligomerization of AIM2^Hin^ is an evolutionarily conserved feature.

### AIM2^PYD^ is required to oligomerize when dsDNA is in excess

**Figure 5:**
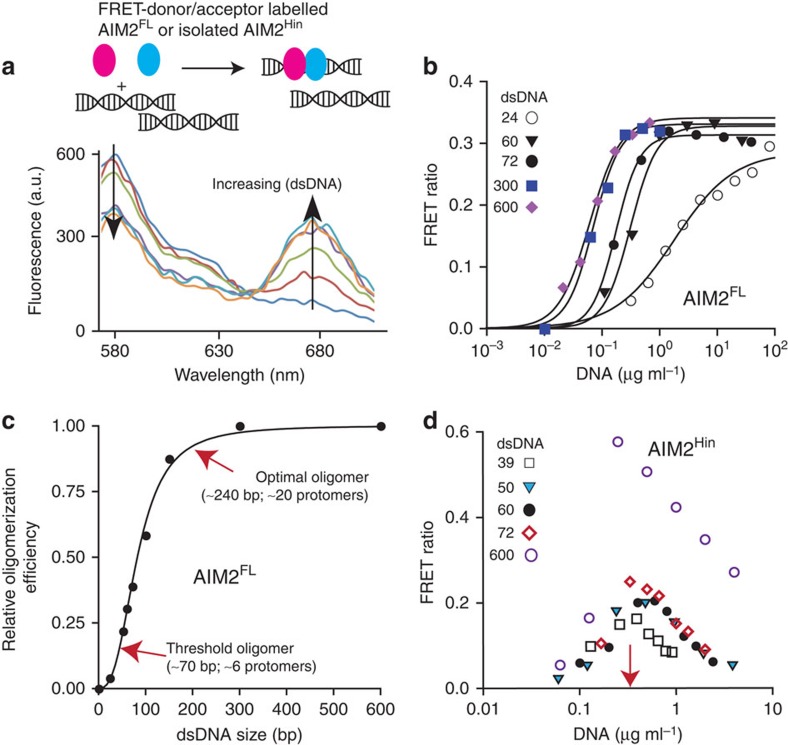
AIM2^PYD^ is necessary for oligomerization and dsDNA binding in the presence of excess dsDNA. (**a**) Top: a cartoon demonstrating the rationale of the described FRET experiments. The two differentially coloured ovals represent fluorophore (Dylight-550 and Dylight-650)-labelled AIM2. Bottom: a sample fluorescence emission spectra of an equimolar mixture of FRET donor and acceptor labelled AIM2FL. (**b**) Changes in the ratio between the FRET donor emission (λmax: 578 nm) and the acceptor emission (λ_max_: 678 nm) at each indicated dsDNA concentration. The apparent oligomerization constants (K_DF_) were obtained by fitting the data to a Hill equation and are listed in Supplementary Table 8. (**c**) A plot of binding efficiency versus the length of dsDNA for AIM2^FL^. The data were fit to the Hill equation (the Hill constant is 3.2±0.3; ±indicates s.d., *n*≥3). The efficiency was determined by normalizing the mean K_DF_ of each fragment to that of dsDNA600. (**d**) The FRET ratio of AIM2Hin with increasing amounts of various dsDNA. The red arrow indicates the concentration of AIM2Hin present in the assay.

When basal AIM2 encounters foreign dsDNA in the cytoplasm, the individual molecules must assemble into the inflammasome even in the presence of excess binding sites (for example, nearly 400,000 binding sites are in the genome of one *F. tularensis*). To test this idea, we labelled two separate AIM2^FL^ populations with a Förster resonance energy transfer (FRET) donor and acceptor (Fig. 5a, top). As previously observed for full-length IFI16 (ref. 23), saturating FRET signals were indeed detected from AIM2^FL^ in a dsDNA size-dependent manner even when the substrate is present in excess (Fig. 5a,b; Supplementary Table 8). However, unlike IFI16 that required at least 60-bp dsDNA^23^, FRET signals were detected from dsDNA fragments as short as 24 bp, suggesting that the minimal binding unit for AIM2 oligomerization is a dimer compared with a tetramer for IFI16. Consistent with our competition assays (Fig. 2), plotting the normalized binding efficiency versus dsDNA length from the FRET assay data also suggests a cooperative relationship in which the binding affinity of AIM2^FL^ can increase as much as 1,000-fold when the size of available dsDNA is 10 times longer (the Hill constant is ∼3; Fig. 5c). Labelled AIM2^Hin^ populations failed to generate FRET signals at 160 mM KCl (Supplementary Fig. 1j,k); however, we detected FRET signals from labelled AIM2^Hin^ if the salt concentration of the reaction buffer was lowered to 60 mM KCl (Fig. 5d). Nevertheless, unlike AIM2^FL^, FRET signals from labelled AIM2^Hin^ also peaked at the dsDNA concentration equivalent to the amount of AIM2^Hin^ present in these assays, but decreased with excess dsDNA (Fig. 5d). Also, unlike AIM2^FL^, the peak amplitude was correlated with the size of each dsDNA (Fig. 5d). These observations suggest that the AIM2^Hin^ oligomers are likely different from those assembled AIM2^FL^, and that AIM2^PYD^ is required for robust dsDNA binding and polymerization in the presence of excess dsDNA. In addition, the observed cooperative relationship between dsDNA size and oligomerization activity (Fig. 5c) is consistent not only with our competition experiments (Fig. 2e) but also with the previous *in vivo* observation^32^, thus further supporting our ‘digital ruler' concept in the regulation of the AIM2 inflammasome.

### *apo*-AIM2^FL^ can auto-oligomerize

**Figure 6:**
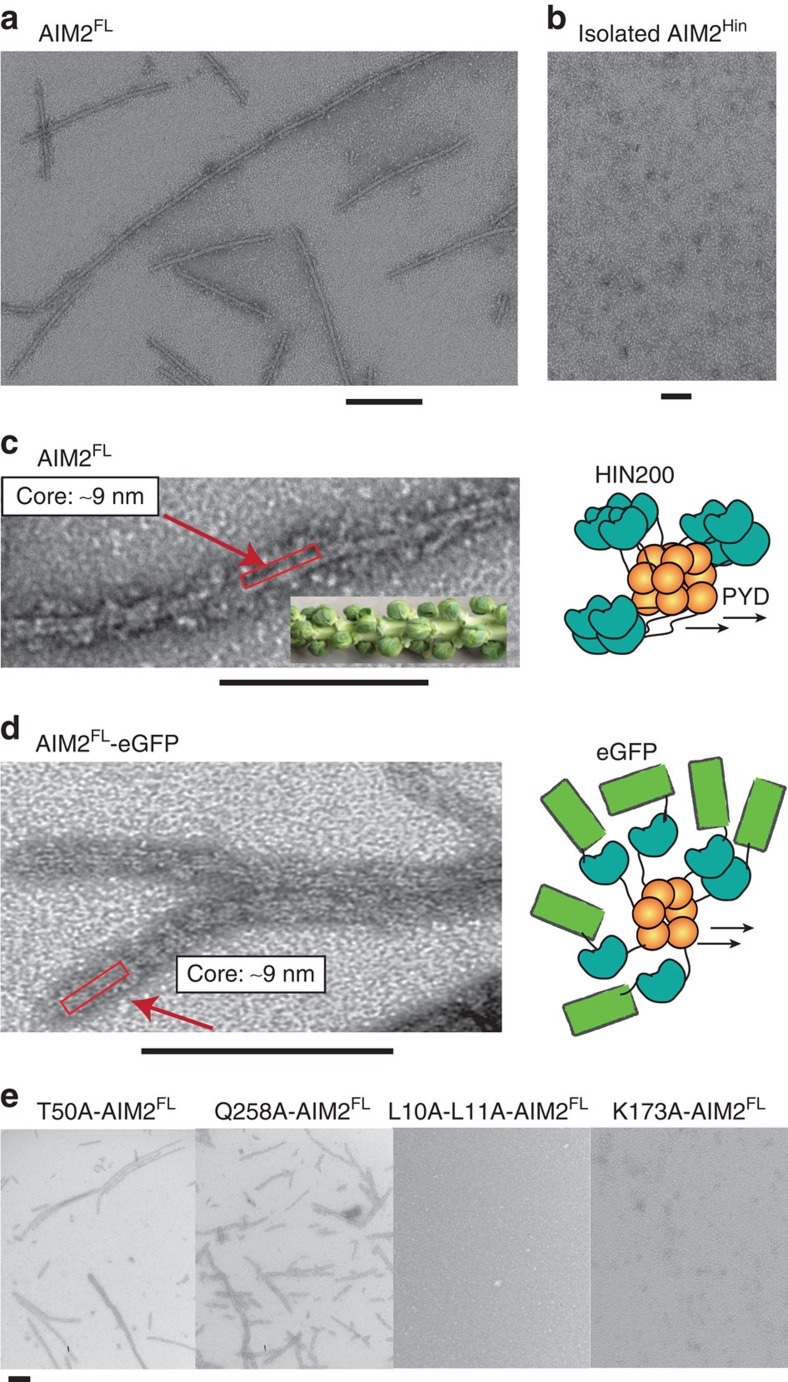
AIM2^FL^ assembles into filaments without dsDNA. (**a**) A negatively stained electron micrograph of AIM2^FL^ at 2 μM. (**b**) A negatively stained electron micrograph of AIM2^Hin^ at 5 μM. (**c**) Higher magnification of the AIM2 filament. The inset is unpicked Brussels sprout, and the cartoon on the right is the proposed overall arrangement of the filament. The red boxes in **c** and **d** indicate the stable ‘core stem' of the AIM2^FL^ filament. (**d**) An electron micrograph of AIM2^FL^–eGFP (2 μM). The cartoon on the right is the proposed overall arrangement of the filament. (**e**) Electron micrographs of AIM2^FL^ mutants at 2 μM. Scale bar, 100 nm.

The autoinhibitory model entails that dsDNA is required to unlock monomeric AIM2 and initiate oligomerization^32^,^33^. By contrast, because untagged AIM2^FL^ binds dsDNA much more tightly than AIM2^Hin^, we hypothesized that the role of dsDNA is to simply increase the local concentration of AIM2 by acting as a ‘one-dimensional ruler,' consequently improving the prospects for forming AIM2^PYD^·AIM2^PYD^ encounter complexes. This new model entails that dsDNA-free AIM2^FL^ should be able to oligomerize on its own if the concentration threshold is met. On the other hand, *apo*-AIM2^FL^ should remain monomeric according to the autoinhibitory model. To test these opposing predictions, we used negative stain electron microscopy (ns-EM) to probe the oligomeric state of *apo*-AIM2^FL^ at various concentrations. Disagreeing with the autoinhibitory model, we found that *apo*-AIM2^FL^ forms filaments in a protein concentration-dependent manner (≥1 μM; Fig. 6a, Supplementary Fig. 2a). By contrast, we did not observe any auto-assembled AIM2^Hin^ filaments (Fig. 6b), and MBP-AIM2^FL^ showed aggregates clearly different from the filamentous structures observed from untagged AIM2^FL^ (Supplementary Fig. 2b). AIM2^FL^ filaments were detected even at 1.6 M NaCl, thus suggesting that they are more resilient against the environment than the ASC^PYD^ filament^7^ (Supplementary Fig. 2c). Considering the auto-oligomerization activity, we kept the concentration of wild-type AIM2^FL^ as low as possible in our biochemical assays (typically <100 nM; Figs 2, 3, 4, 5). Moreover, we observed saturating binding isotherms in most of our assays (Fig. 2a), suggesting that auto-oligomerization did not cause any significant artifacts.

Closer inspection of the electron micrographs revealed that DNA-free AIM2^FL^ filaments assume ‘Brussels Sprout'-like structures in which the central filaments (‘core stems') are decorated with speck-like densities at the periphery (‘sprouts') (Fig. 6c, Supplementary Fig. 2c,d). Although the core stem appeared well ordered, the peripheral specks appeared random and often disordered (Fig. 6c, Supplementary Fig. 2c,d). On the basis of the reported auto-filamentation activity of isolated AIM2^PYD^ (refs 7, 34), we suspected that AIM2^PYD^ forms the core stem and the AIM2^Hin^ clusters are flexibly attached via the intrinsically disordered linker region (50 amino acids); the 9-nm diameter of the core stem also corresponds to the width of the ASC^PYD^ filament^7^. To further test this arrangement, we generated an AIM2^FL^ construct with enhanced green fluorescent protein (eGFP) fused on the C-terminus; we added eGFP at the C-terminus so that it would not directly interfere with the putative oligomeric structure of AIM2^PYD^ (Fig. 6d, cartoon). The electron micrograph of AIM2^FL^–eGFP also showed similar filament morphology as untagged AIM2^FL^ where the core stem is randomly decorated by peripheral densities (Fig. 6d). The peripheral densities were also more apparent than those from untagged AIM2^FL^, likely reflecting the added size of eGFP.

### dsDNA-binding deficient mutants fail to auto-oligomerize

If the mutations we have identified in the present study indeed affect oligomerization, but not direct protein–dsDNA interactions, we reasoned that they should also disrupt the auto-filamentation activity. Hence, we examined these AIM2 mutants using ns-EM. The Brussels sprout-like filaments were detected from the AIM2^FL^ mutants that lacked any major defects in dsDNA binding (Fig. 6e and Supplementary Fig. 3a,b). However, the AIM2^FL^ mutants with impaired dsDNA binding did not show any auto-assembled filaments regardless of whether a mutation is located on the PYD or HIN200 domains (Fig. 6e, Supplementary Fig. 3a). The failure to auto-assemble by mutating the PYD side chains suggests that it is the filamentation activity of AIM2^PYD^ that positively contributes to dsDNA binding. Moreover, the lack of auto-oligomerization resulting from mutating the HIN200 domain side chains suggests that although the oligomerization activity of AIM2^PYD^ outweighs that of AIM2^Hin^, both domains have positive functions in assembling AIM2^FL^ filaments in the presence or absence of dsDNA.

### Helical symmetry of the AIM2^PYD^ filament

**Figure 7:**
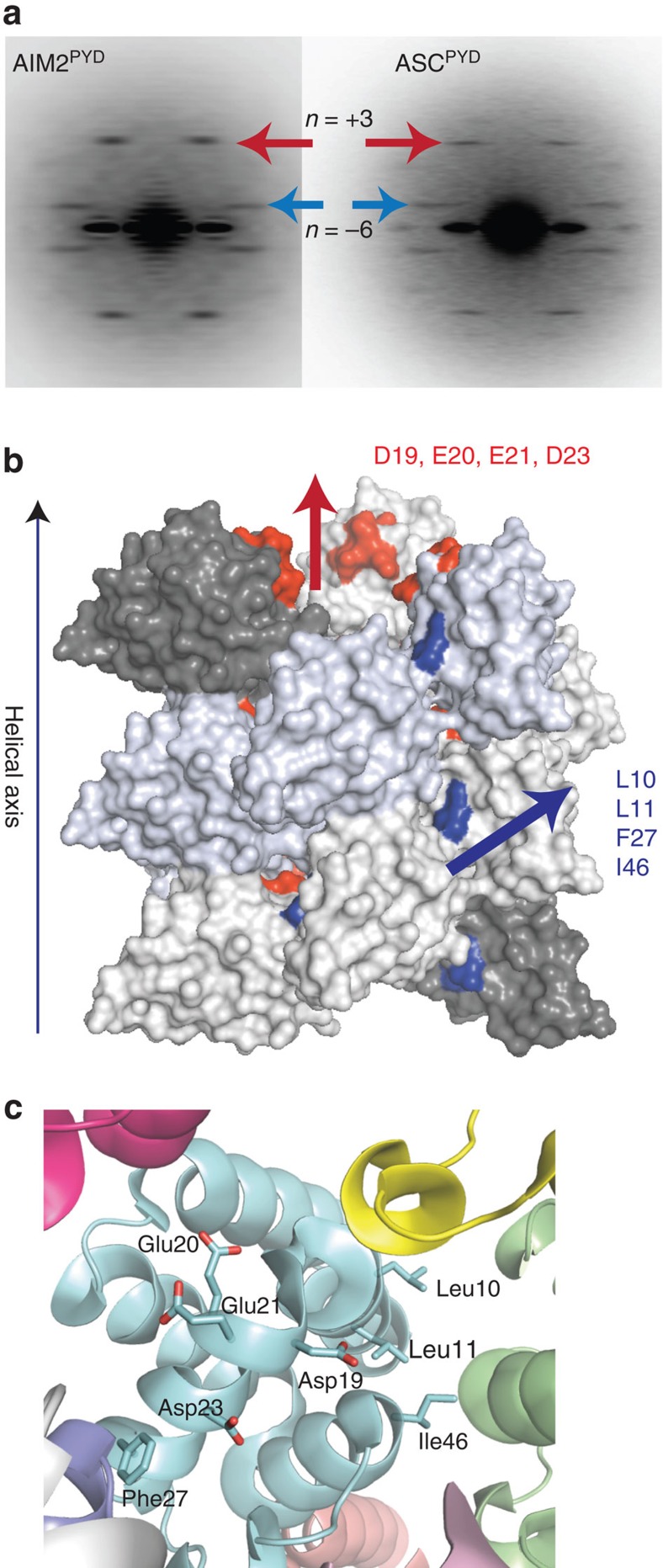
The congruent helical symmetry between filaments assembled by AIM2^PYD^ and ASC^PYD^. (**a**) The ns-EM average power spectra of the AIM2^PYD^ filament (left) and the ASC^PYD^ filament (right; personal communication; Dr Hao Wu, Harvard). The coloured arrows indicate corresponding helical symmetry lines observed from both filaments. (**b**,**c**) A homology of model of the AIM2^PYD^ filament based on the cryo-EM structure of the ASC^PYD^ filament (PDB ID: 3J63). The AIM2 side chains important for dsDNA binding and auto-oligomerization are highlighted.

Several previous studies reported the direct interaction between AIM2^PYD^ and ASC^PYD^ (refs 7, 19, 20). The helical architecture of the ASC^PYD^ filament is known^7^; however, whether or not the upstream AIM2 oligomer provides a ‘polymerization template ' via a congruent oligomeric architecture as seen from the Rig-I·MAVS interaction^9^ is an open question. Thus, we determined the helical symmetry of the core stem of the AIM2^FL^ filaments. The average power spectrum of the AIM2^FL^ filaments obtained from ns-EM is remarkably similar to that of the ASC^PYD^ filament (Fig. 7a, see also ref. 7), which is an ∼90-Å wide, six-start helix with three-fold symmetry^7^. These parameters also agree with our proposition in which the AIM2^PYD^ filament constitutes the core stem (Fig. 6). On the basis of the consistent helical symmetry, we generated a homology model of the AIM2^PYD^ filament using the cryo-EM structure of the ASC^PYD^ filament as a template^7^, where we found that all the side chains we have identified to be important for dsDNA binding and auto-oligomerization are located at the subunit interfaces (Fig. 7b,c). Overall, our EM analysis suggests that the upstream AIM2^PYD^ filament provides a structural template for the polymerization of downstream ASC^PYD^ (Fig. 1a).

### PYD interactions dictate the filament architecture

**Figure 8:**
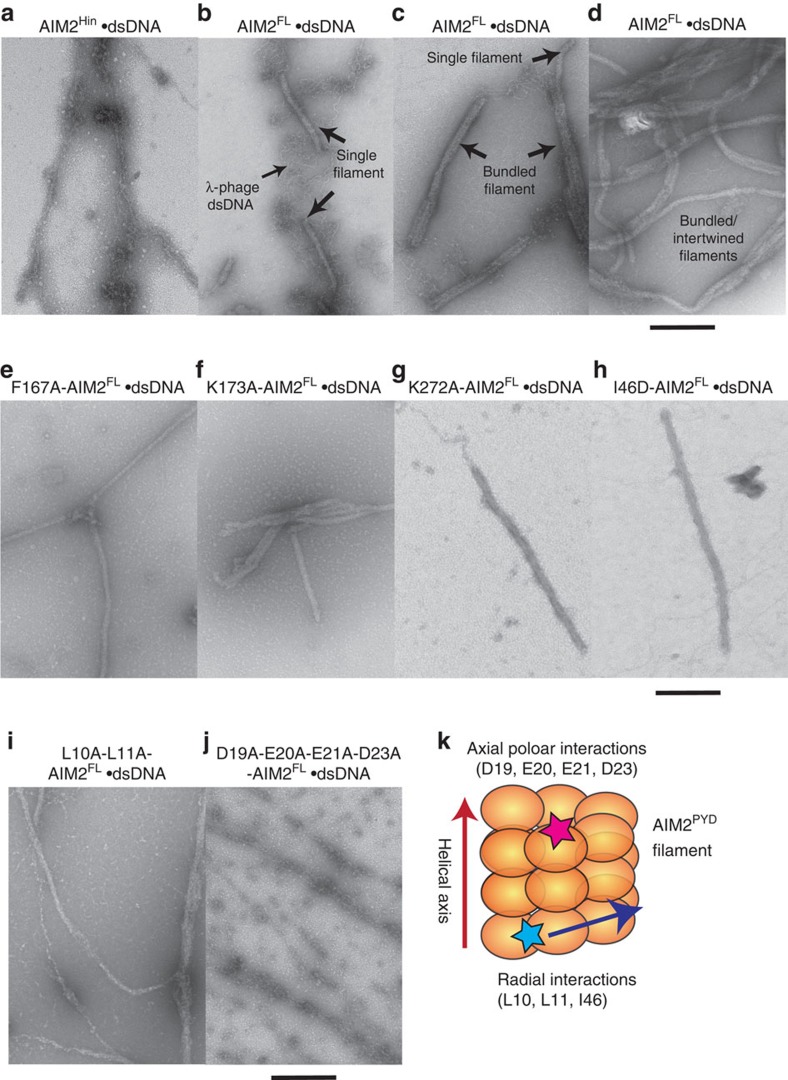
AIM2^PYD^ is required to assemble filamentous structures on dsDNA. (**a**) An electron micrograph of AIM2^Hin^ clusters on λdsDNA (**b–d**) Electron micrographs of wild-type AIM2^FL^ filaments assembled on λdsDNA. (**e–j**) Electron micrographs of AIM2^FL^ mutants bound to λdsDNA. (**k**) A cartoon of the AIM2^PYD^ filament and the locations of mutated side chains based on the congruent helical symmetry between AIM2^PYD^ and ASC^PYD^ (see also Fig. 7b). Scale bar, 100 nm.

A previous cell-based imaging study showed that isolated AIM2^PYD^ and AIM2^FL^ form filamentous aggregates, but AIM2^Hin^ failed to form such structures^34^; whether dsDNA (transfected plasmid) is part of the filamentous AIM2^FL^ aggregates is unknown. Our present study is consistent with this *in vivo* observation^34^, as dsDNA-free AIM2^FL^ assembles into filaments via its PYD. However, in principle, the HIN200 domains of AIM2^FL^ could bind dsDNA along its length, and thus might also generate an ordered filamentous structure. Thus, to further resolve whether AIM2^PYD^ or AIM2^Hin^ oligomers dictate the overall architecture of dsDNA-bound AIM2^FL^, we determined the morphologies of AIM2^FL^ and AIM2^Hin^ bound to λ-phage dsDNA (λdsDNA) using ns-EM. Isolated AIM2^Hin^ did not show any ordered filaments, but displayed random ‘beads on a string'-like clusters on λdsDNA (Fig. 8a). By contrast, the Brussels sprout-like filaments were no longer detected on adding dsDNA to AIM2^FL^, but new larger filaments about two- to three-times wider than the DNA-free filaments appeared in the micrographs (20–25 nm; Fig. 8b), indicating dsDNA binding. Together, these observations suggest that AIM2^Hin^ binds dsDNA and clusters randomly, and that AIM2^PYD^ oligomers underpin the filamentous architecture of dsDNA-bound AIM2^FL^.

λdsDNA is about 50 kb and displayed random-coil structures (Fig. 8b, Supplementary Fig. 3c); however, all observed dsDNA-bound AIM2^FL^ filaments seemed well ordered, further suggesting that PYD–PYD interactions, but not AIM2^Hin^–dsDNA interactions (Fig. 8b), dictate the overall architecture of λdsDNA-bound AIM2^FL^ filaments. Because we can observe unbound λdsDNA in our AIM2^FL^·dsDNA samples (Fig. 8b), our EM experiments also revealed that AIM2^FL^ filaments assemble from random positions on dsDNA and that the filaments can assemble even in the presence of excess dsDNA (Fig. 8b). These results not only corroborate our FRET assays in which AIM2^FL^ oligomerized in the presence of excess dsDNA (Fig. 5) but also are consistent with the oligomerization activity of IFI16, which also formed filaments in the presence of excess dsDNA via its PYD^23^. Several filaments apparently merged laterally and became intertwined, likely reflecting the punctate-like AIM2 inflammasome structures observed from *in vivo* studies^20^,^32^ (Fig. 8c,d). In addition, we used supercoiled and nicked circular plasmids to investigate whether the morphology of AIM2^FL^ filaments can be altered by the structure of the dsDNA platform. The AIM2^FL^ filaments assembled on these nonlinear dsDNA molecules were isomorphic to the λdsDNA-bound AIM2^FL^ filaments, also suggesting that protein–protein interactions indeed dominate the overall architecture of the AIM2^FL^·dsDNA complexes (Supplementary Fig. 3d).

To further test that AIM2^PYD^ oligomers, but not those assembled by AIM2^Hin^, underpin the filamentous structure of dsDNA-bound AIM2^FL^, we also examined the morphology of several λdsDNA-bound AIM2^FL^ mutants with defective dsDNA-binding/auto-assembly activities. We first identified conditions that allow these AIM2^FL^ mutants to bind dsDNA (160 mM KCl; same as all wild-type EM experiments); all the defective mutants still bound dsDNA significantly more weakly than wild type (Supplementary Table 1). Under these conditions, we found that disrupting the oligomerization activity of AIM2^Hin^ still allowed the full-length protein to assemble into isomorphic filaments on λdsDNA as wild type (Fig. 8e–g). The I46D mutation located in the PYD also resulted in wild type-like AIM2^FL^ filaments once bound to λdsDNA (Fig. 8h). However, L10A-L11A-AIM2^FL^ showed a heterogeneous nucleoprotein filament population (Fig. 8i). More strikingly, D19A-E20A-E21A-D23A-AIM2^FL^ (acidic patch) displayed disordered clusters similar to λdsDNA-bound isolated AIM2^Hin^ (Fig. 8j). Our homology model indicates that Leu10, Leu11 and Ile46 mediate the radial interactions in the AIM2^PYD^ filament, while the acidic patch mediates the axial interactions (Figs 7b and 8k). Thus, these results suggest that the dsDNA scaffold can at least partially restore the filamentous structure if the radial interactions in the AIM2^PYD^ filament are compromised (Figs 7b and 8k). However, disrupting the axial interactions seems to be detrimental to the filamentous structure even when bound to the dsDNA scaffold (Figs 7b and 8k). Overall, we concluded that filamentous AIM2^PYD^ oligomers underpin the architecture of dsDNA-bound AIM2^FL^ polymers.

## Discussion

**Figure 9:**
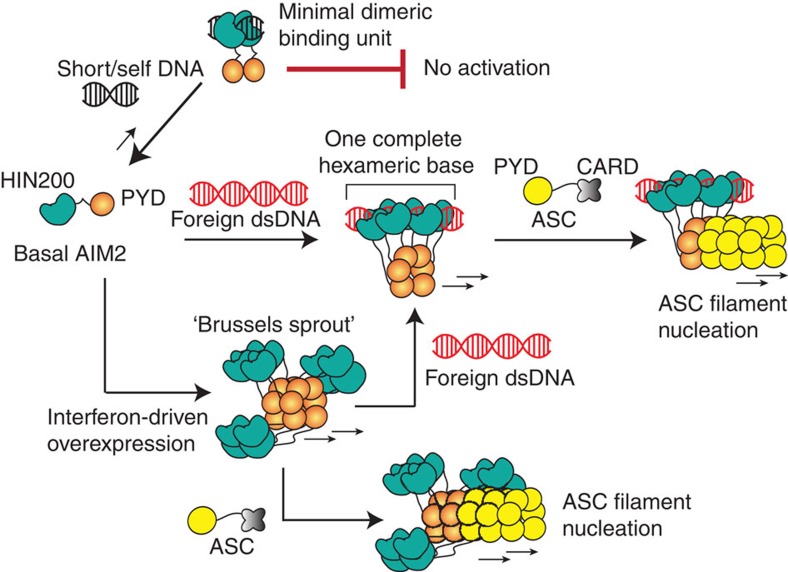
A model for the assembly of the AIM2 inflammasome. AIM2^PYD^ is not auto-regulated. Depending on its cellular concentration, auto-assembly or dsDNA-mediated assembly will drive the initial filament formation. Importantly, basal AIM2 requires large dsDNA to generate energetically stable nucleoprotein complexes, because oligomerization is integral to dsDNA binding. The oligomerization of AIM2^Hin^ is important for auto- or dsDNA-mediated filament assembly, but the construction of the filamentous architecture is dictated by AIM2^PYD^. The resulting AIM2 filaments then nucleate the assembly of the ASC filaments via corresponding helical architecture (ASC^CARD^ is not shown in the filament for simplicity).

We have presented here evidence supporting an oligomerization-driven activation mechanism for initiating the assembly of the AIM2 inflammasome in the absence of the currently prevailing autoinhibitory mechanism^32^,^33^ (Fig. 9). For instance, the basal concentration of AIM2 is presumably sub-nanomolar under normal conditions, but is dramatically raised on pathogenic invasion (AIM2 is overexpressed by type-1 interferons by at least 200-fold)^18^,^20^,^39^. Thus, in the absence of cytosolic dsDNA, we propose that AIM2 would fail to oligomerize and induce the polymerization of ASC, due to its low basal concentration. For instance, in physiologically relevant reaction conditions, AIM2 can assemble into a filament on dsDNA larger than 300 bp even at pico-molar concentrations, but it requires nearly 10,000-fold more AIM2 molecules to auto-assemble filaments without dsDNA (Figs 2 and 5, and Supplementary Fig. 2a). On the other hand, because oligomerization is coupled with dsDNA binding, individual AIM2 molecules are capable of finding one another even in the presence of excess dsDNA (Fig. 5). Thus, when foreign dsDNA invades, basal AIM2 would rapidly cluster into a seed filament necessary to nucleate the polymerization of ASC.

The assembly of Rig-I and MDA5 filaments on foreign dsRNA is intrinsically regulated by the ATP turnover at their helicase domains^28^,^29^,^30^; the filament assembly of Rig-I is further regulated by the recognition of 5′-triphosphate of dsRNA^1^,^30^. However, ALRs lack any dsDNA sequence specificity and auto-assemble into filaments without any cofactors^31^. These observations raise the question of whether ALRs are regulated at all. We propose that the dsDNA length-dependent binding provides a key to answering this question. Although MDA5 can assemble into filaments along the length of dsRNA, the ability to discriminate between ‘short' and ‘long' dsRNA by MDA5 is not as pronounced as that of AIM2 or IFI16 (refs 23, 28), as if separating the ligand-binding domain from the polymerization domain generates greater dependence on the size of the nucleic acid scaffold for assembly. ‘Long,' naked cytosolic dsDNA is rare, and Knipe and colleagues postulated that IFI16 would selectively recognize foreign dsDNA by the degree of chromatinization^12^,^40^,^41^. Indeed, the highly cooperative relationship between the binding affinity and the size of dsDNA can clearly define an ‘off' and ‘on' state for assembling the AIM2 nucleoprotein filament (Figs 2 and 5). Moreover, the size of dsDNA required to build the ‘threshold oligomer' (Figs 2 and 5) also correlates with a previous *in vivo* study in which about 80-bp cytosolic dsDNA was required to induce robust interleukin-1β secretion^32^. Taken together, our data suggest that the size of dsDNA can act as a powerful ‘molecular ruler' that can regulate the initiation of the AIM2 inflammasome assembly in a switch-like mechanism.

Unlike the HIN200 domains of IFI16 (ref. 23), we found that AIM2^Hin^ oligomerizes on dsDNA, which appears to be at least partially responsible for the more robust dsDNA-binding activity of AIM2 than IFI16 *in vitro*^23^,^32^. It is tempting to speculate that the oligomerization of AIM2^Hin^ has not been selected against because cytosolic AIM2 is much less likely to encounter self-dsDNA than nuclear IFI16. On the other hand, p202^HinB^ does not bind dsDNA, but forms a tetramer that prevents AIM2 from clustering on dsDNA^38^. Our finding of the oligomerization activity of AIM2^Hin^ further strengthens this finding, as p202^HinB^ would physically interfere with clustering of AIM2^Hin^ on dsDNA. Moreover, not all the side chains implicated in the tetramerization of p202^HinB^ are conserved in AIM2 (ref. 38; Fig. 4a,b), thus preventing spurious auto-oligomerization. Nevertheless, our experiments showed that isolated AIM2^Hin^ oligomerizes into random clusters on dsDNA without any filamentous architecture (Fig. 8a) and that disrupting the oligomerization of AIM2^Hin^ still results in filaments isomorphic to wild type (Fig. 8e–g). Thus, we hypothesize that the role of AIM2^Hin^ oligomerization is essential, yet limited to generating a ‘seed' nucleation unit preceding the filament growth (Fig. 9).

AIM2 is overexpressed by type-1 interferon pathways on pathogenic invasion^31^,^39^. Our finding of auto-assembly suggest that in principle, dsDNA binding is not *a priori* required to initiate assembly. We hypothesize that the auto-oligomerization of AIM2 (Fig. 6) can also enhance the host defence response (Fig. 9). For example, the pre-assembled AIM2 platforms would be able to survey the cytoplasm more effectively because of the larger contiguous surface area. The AIM2^Hin^ clusters could also increase the dsDNA-binding activity via avidity, and the AIM2 oligomers would immediately nucleate the polymerization of ASC without binding foreign dsDNA. However, this could be a double-edged sword: the auto-oligomerization activity of AIM2 could also underlie several autoimmune disorders in which AIM2 is overexpressed via hyperactive interferon pathways^14^,^15^,^16^, as it could cause persistent inflammasome activity without any pathogenic dsDNA.

The symmetry of the AIM2 filament suggests the structure–activity relationships in the assembly. For instance, the three-fold symmetry of the six-start helix correlates with the dimeric minimal binding unit of AIM2 observed from our FRET assays (Fig. 5b,c; 2 × 3=6). The requirement for clustering about six AIM2 molecules (Figs 2e and 5c) to generate a robust ‘threshold oligomer' also correlates with one hexameric base of the six-start helix. Furthermore, the optimal oligomer size of 20–25 AIM2 molecules (Figs 2e and 5c) also suggests that about four hexameric rings may need to stack up to assemble an optimally stable AIM2 nucleoprotein filament. Finally, because the ASC^PYD^ filament also has the same helical architecture as the AIM2^PYD^ filament, it is tempting to speculate that the assembly of the upstream filament is directly coupled to the downstream effector activation, thus achieving maximal cooperativity.

Both PYDs and CARDs belong to the death-domain (DD) superfamily^36^,^42^. Despite their widely variable primary sequences, all known CARDs and PYDs share essentially the same tertiary structure (six-helix bundles)^36^,^42^. Several DD proteins are capable of assembling into helical filaments^7^,^9^,^43^, and the PYDs are mostly distinguished from the CARDs by one extended loop region between helices 2 and 3 (refs 36, 42). Thus, a major outstanding question regarding the signalling mechanism of both PYDs and CARDs has been how one DD protein specifically selects its interacting partner. Although all DD proteins utilize essentially the same set of interaction surfaces for oligomerization^36^,^42^, it is becoming clear that the resulting oligomers display vastly diverse helical architectures^7^,^9^,^43^. Indeed, it was recently shown that identical helical symmetry underlies the mechanism by which the Rig-I^CARD^ tetramers nucleate the MAVS^CARD^ filament^9^. It had yet to be tested whether such a symmetric interaction is a unifying theme in assembling filamentous supramolecular signalling platforms by both CARDs and PYDs, especially when both upstream and downstream DD domains can assemble into infinite filaments as observed from AIM2 and ASC. The consistent helical symmetry between the AIM2^PYD^ and ASC^PYD^ filaments suggests that the corresponding polymerization trajectory between the upstream and downstream oligomers not only underpins the assembly of the inflammasomes but also can be a key to defining the specificity of the DD-family proteins.

## Methods

### Reagents

All DNA below 90 bp were purchased from Integrated DNA Technologies, and DNA of greater length was synthesized by PCR. FAM-labelled DNA was also purchased from Integrated DNA Technologies. DyLight-550 and DyLight-650 maleimides were purchased from Thermo.

### Recombinant AIM2 constructs

Full-length AIM2 variants (residues 1–343) were cloned into a pET21 vector (Novagen) with a modified N-terminal MBP tag with a TEV protease recognition site. HIN200 variants (residues 144–343) were cloned into a pET28 vector with a modified N-terminal small ubiquitin-like modifier (SUMO) protein tag or the previously described pET21 vector for assays including an MBP tag. All clones were transformed into *Escherichia coli* strain ER2566 (NEB). Cells were grown at 37 °C to OD_600_ 0.4–0.7, induced with 0.2 mM isopropyl β-D-1-thiogalactopyranoside, expressed for at least 15 h at 18 °C, and then harvested.

For purification of unlabelled protein, cell pellets were resuspended in 20 mM HEPES pH 7.4, 400 mM NaCl, 5% glycerol, 1 mM EDTA and 1 mM dithiothreitol (DTT) while SUMO-tagged construct cell pellets were resuspended in 20 mM HEPES pH 7.4, 400 mM NaCl, 5% glycerol, 20 mM imidazole and 3 mM beta-mercaptoethanolamine (βME); a protease cocktail consisting of phenylmethyl sulfonyl fluoride, benzamidine, leupeptin and pepstatin A was added, as well as lysozyme and DNase I. Cells were lysed by sonication and the insoluble fraction was removed by centrifugation. The supernatants were applied to amylose resin (NEB) for MBP-tagged constructs or Ni-NTA resin (QIAgen) for SUMO-tagged constructs. A wash of 10–15 CV of resuspension buffer was applied, and then the protein was eluted in either 20 mM HEPES pH 7.4, 120 mM NaCl, 2% glycerol, 30 mM maltose and 3 mM βME for MBP-tagged or 20 mM HEPES pH 7.4, 120 mM NaCl, 2% glycerol, 300 mM imidazole and 3 mM βME for SUMO-tagged constructs. The elution was then applied to a HiTrap-SP Column (GE Healthcare) and the protein eluted off a gradient of 20 mM HEPES pH 7.4, 120 mM–1M NaCl, 2% glycerol and 3 mM βME. Fractions containing highly purified protein were collected. For biochemical assays using MBP-tagged protein, these fractions were then applied to a Superdex200 16/600 gel-filtration column (GE Healthcare) equilibrated in 20 mM HEPES pH 7.4, 400 mM KCl, 2% glycerol, 1 mM EDTA and 1 mM DTT. For biochemical assays using untagged protein, the pooled fractions were diluted 10-fold with 20 mM HEPES pH 7.4, 750 mM NaCl, 2% glycerol, 20 mM imidazole and 3 mM βME, and TEV (MBP tag) or Ulp-1 (SUMO tag) was added, and the solution was dialysed at least 15 h against 20 mM HEPES pH 7.4, 120 mM NaCl, 2% glycerol, 20 mM imidazole and 3 mM βME. For full length, the solution was then re-applied to the HiTrap-SP column, and then eluted off in tandem with a HisTrap-FF (GE Healthcare) with 20 mM HEPES pH 7.4, 750 mM NaCl, 2% glycerol, 20 mM imidazole and 3 mM βME. For HIN200, the solution was applied to a HisTrap-FF column in tandem with a HiTrap-SP column, the HisTrap column was then removed and the protein was eluted from the HiTrap column in the same buffer as the full length. The elution fractions were applied directly to a Superdex75 16/600 gel-filtration column (GE Healthcare) equilibrated in 20 mM HEPES pH 7.4, 400 mM KCl, 2% glycerol, 1 mM EDTA and 1 mM DTT. Fractions containing the protein were then concentrated.

For fluorescent labelling of proteins, after elution from their respective affinity columns, they were applied to a HiTrap-SP column and eluted using a gradient of 20 mM HEPES pH 7.4, 120 mM NaCl–1M NaCl, 2% glycerol and 1 mM TCEP. The solution was then divided into two, and to one was added DyLight-550 at a concentration of 4 × (AIM2) and to the other was added DyLight-650 at a concentration of 4 × (AIM2). Labelling proceeded for at least 15 h. Excess dye was quenched using 10 mM βME, and the solution was re-applied to their respective affinity columns. After extensive washing with 20 mM HEPES pH 7.4, 750 mM NaCl, 2% glycerol and 3 mM βME until no fluorescence could be detected in the flow through, the protein was eluted in wash buffer supplemented with either 30 mM maltose (full length) or 300 mM imidazole (HIN200). Cleavage and further purification proceeded as above. The dye:protein ratio was then calculated as per the manufacturer's instructions, which was ∼1:1.

### Biochemical assays

All experiments were performed at least three times, the fits to data were generated by the Kaleidagraph software (Synergy). Fluorescence anisotropy-binding experiments were carried out in either 40 mM HEPES pH 7.4, 160 mM KCl, 5% glycerol, 0.1% Triton X-100, 1 mM EDTA, 5 mM DTT (herein referred to as ‘buffer A') or 40 mM HEPES pH 7.4, 400 mM KCl, 5% glycerol, 0.1% Triton X-100, 1 mM EDTA and 5 mM DTT (herein referred to as ‘buffer B') at room temperature as described in Morrone *et al.*^23^

Förster resonance energy transfer experiments were carried out in either buffer A (for AIM2^FL^ and AIM2^Hin^) or buffer C (40 mM HEPES pH 7.4, 60 mM KCl, 5% glycerol, 0.1% Triton X-100, 1 mM EDTA and 5 mM DTT) for AIM2^Hin^ as described in Morrone *et al.*^23^

Electrophoretic mobility shift assay experiments were carried out in buffer A. To a fixed amount of fluorescein-labelled dsVACV72 was added increasing concentrations of AIM2. The reaction was allowed to equilibrate at room temperature (at least 20 min), then applied to a 4% 116:1 acrylamide:bis-acrylamide Tris-borate-EDTA gel. The gel was run at 100 V in 1 × Tris-borate-EDTA buffer and imaged using a Typhoon imager (GE Healthcare; excitation at 488 nm, emission at 532 nm).

### Electron microscopy

AIM2 samples were adsorbed to glow-discharged carbon grids for 2 min, then blotted and transferred through two consecutive drops of 1% uranyl formate or 1% uranyl acetate for a total of 1–2 min. The carbon film was then quickly dried by aspiration. Images were collected with either a Philips BioTwin CM120 (FEI) at Johns Hopkins School of Medicine or Tecnai 12 at University of Virginia. For the dsDNA·AIM2 complexes, λdsDNA and AIM2 constructs were incubated 30 min before EM sample preparation. The linearized plasmid was generated by digesting the pET28b vector (Novagen) with BamH1 (NEB). The nicked circle was generated by cutting pET28b with Nt.BspQI (NEB). The modified vectors were then agarose-gel purified.

### Homology modelling

The crystal structure of AIM2^PYD^ (PDB ID: 3VD8) was aligned to individual protomers of the cryo-EM structure of the ASC^PYD^ filament (PDB ID: 3J63) using Pymol (The PyMOL Molecular Graphics System, Version 1.7.4 Schrödinger, LLC). The root mean squared deviation of the individual alignment is <1 Å.

### Symmetry determination

Micrographs of negatively stained AIM2^FL^ filaments were scanned using a Nikon CoolPix 8000 with a raster of 4.16 Å/pixel. Filaments were extracted using the e2helixboxer routine within EMAN2 (ref. 44), and the SPIDER software package^45^ was used for subsequent steps. Overlapping boxes 96 pixels long were cut from these filaments, and 7,607 boxes were aligned against a preliminary reconstruction and windowed to 30 pixels to generate an averaged power spectrum.

## Additional information

**How to cite this article:** Morrone, S. R. *et al.* Assembly-driven activation of the AIM2 foreign-dsDNA sensor provides a polymerization template for downstream ASC. *Nat. Commun.* 6:7827 doi: 10.1038/ncomms8827 (2015).

## Supplementary Material

Supplementary Information Supplementary Figures 1-3 and Supplementary Tables 1-8
