# Supplementary material for: Assembly-driven activation of the AIM2 foreign-dsDNA sensor provides a polymerization template for downstream ASC
Source: Nat Commun. 2015 Jul 22;6:7827. doi: 10.1038/ncomms8827 (PMC4525163; doi:10.1038/ncomms8827)
Supplement: Supplementary Information — Supplementary Figures 1-3 and Supplementary Tables 1-8 [file ncomms8827-s1.pdf]

Supplementary Fig. 1

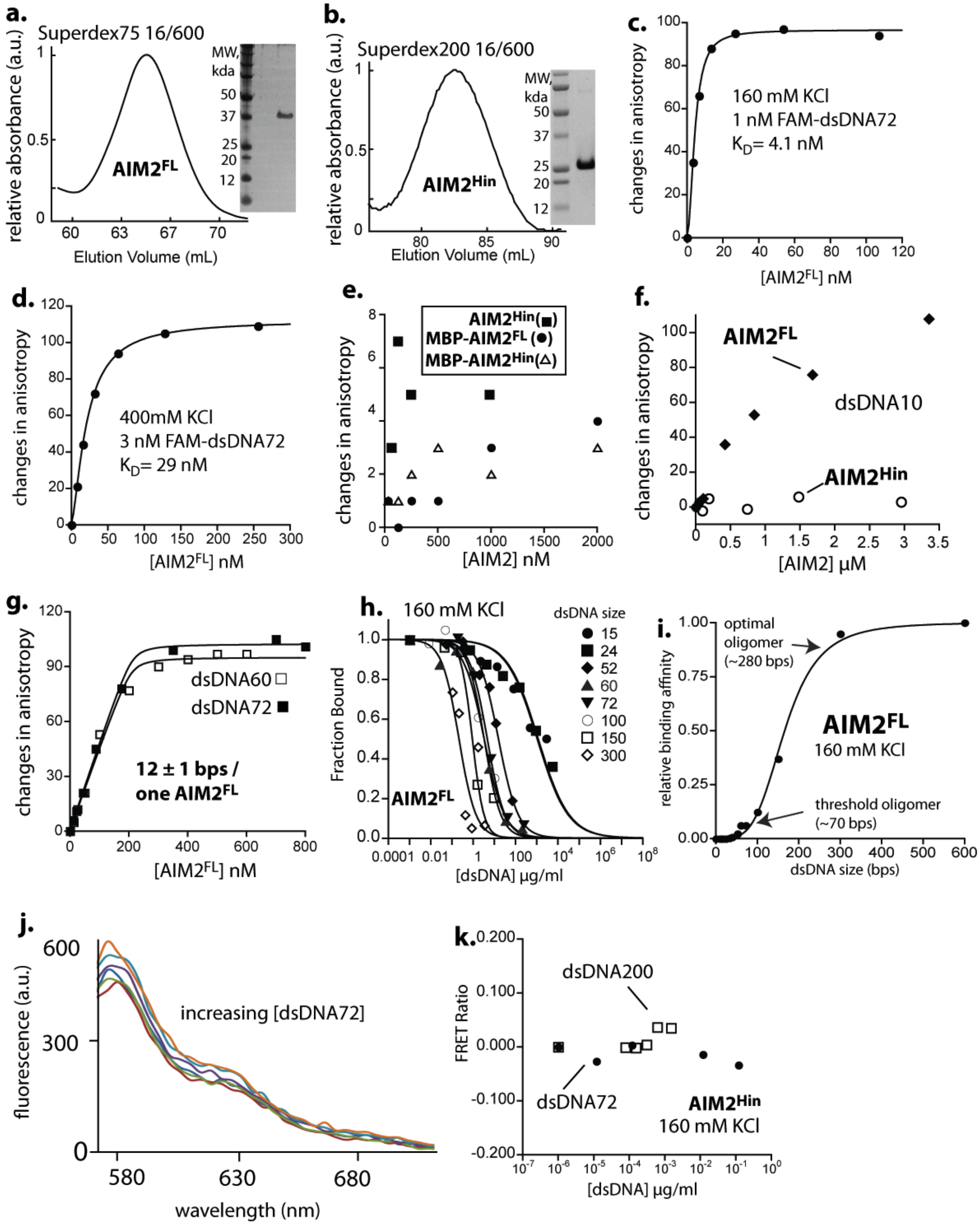

Supplementary Fig. 1. Biochemical properties of AIM2

(**a**) Gel-filtration profiles and SDS-PAGE images of AIM2^{FL} and (**b**) AIM2^{Hin}. The gels were silver-stained for the visualization of AIM2 constructs. (**c**) Binding of AIM2^{FL} toward 1 nM FAM-

dsDNA72 at 160 mM KCl. Compared to the experiment described in Fig. 1b, the calculated binding affinity decreased by two-fold (4 vs. 8 nM in Fig. 1b) by lowering the labeled dsDNA concentration by about two-fold (1 vs. 2.5 nM in Fig. 1b). (**d**) Binding of AIM2^{FL} toward 3 nM FAM-dsDNA72 at 400 mM KCl. Unlike at 160 mM KCl (i.e. Fig. 1b vs. Supplementary Fig. 1c), the apparent binding affinity did not increase despite raising the labeled dsDNA concentration by two-fold from the experiment described in Fig. 2a (1.5 nM). (**e**) Binding of AIM2 variants toward 3 nM FAM-dsDNA72 at 400 mM KCl. (**f**) Binding of AIM2^{FL} and AIM2^{Hin} toward FAM-dsDNA10 (5 nM) was assayed at 160 mM KCl. (**g**) The footprint of AIM2^{FL} was determined by the stoichiometry titration experiments using 40 nM of FAM-dsDNA60 and FAM-dsDNA72 at 160 mM KCl. The inflection points were 210 and 213 nM AIM2^{FL} for dsDNA60 and dsDNA72, respectively. (**h**) Competition binding assays of AIM2^{FL}•FAM-dsDNA72 (250 and 5 nM, respectively) against various dsDNA at 160 mM KCl. The determined K_D values are listed in Supplementary Table 5. (**i**) The relative binding efficiency of AIM2^{FL} with respect to the size of dsDNA from Supplementary Fig. 1e was determined as described in Fig. 2c. Sample fluorescence emission spectra of FRET donor and acceptor labeled AIM2^{Hin} (25 nM each) with increasing dsDNA concentrations (**j**), and determined FRET ratio at each dsDNA concentration (**k**).

Supplementary Fig. 2

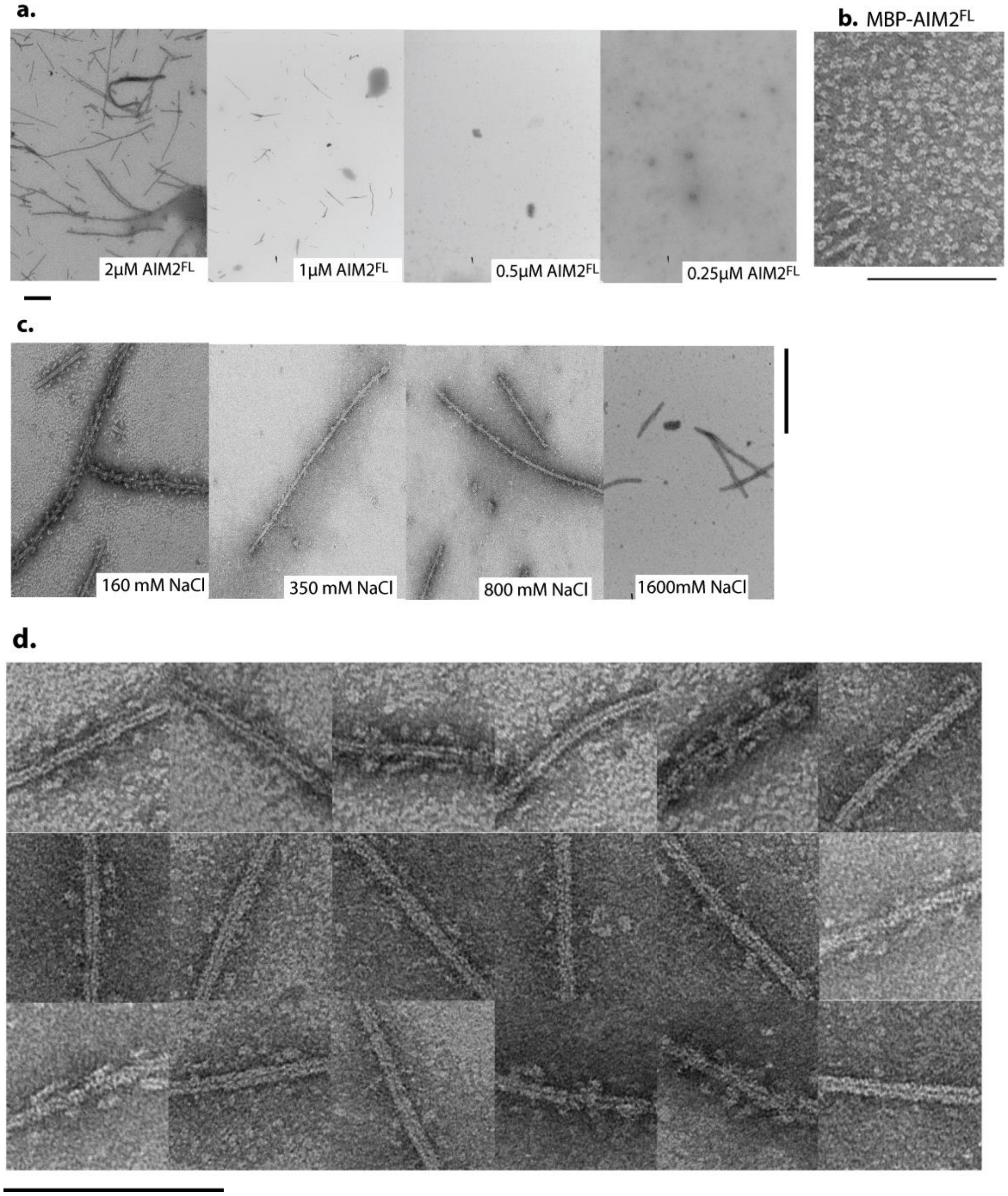

Supplementary Fig. 2. EM studies of wild-type AIM2^{FL}

(**a**) Electron micrographs of AIM2^{FL} at various concentrations. The scale bar is 500 nm. (**b**) An electron micrograph of MBP-AIM2^{FL} (2 μM). The scale bar is 100 nm. (**c**) Electron micrographs of AIM2^{FL} (2 μM) at various salt concentrations. The scale bar is 100 nm (**d**) Close-up views of the dsDNA-free AIM2^{FL} filaments. The scale bar is 100 nm

Supplementary Fig. 3

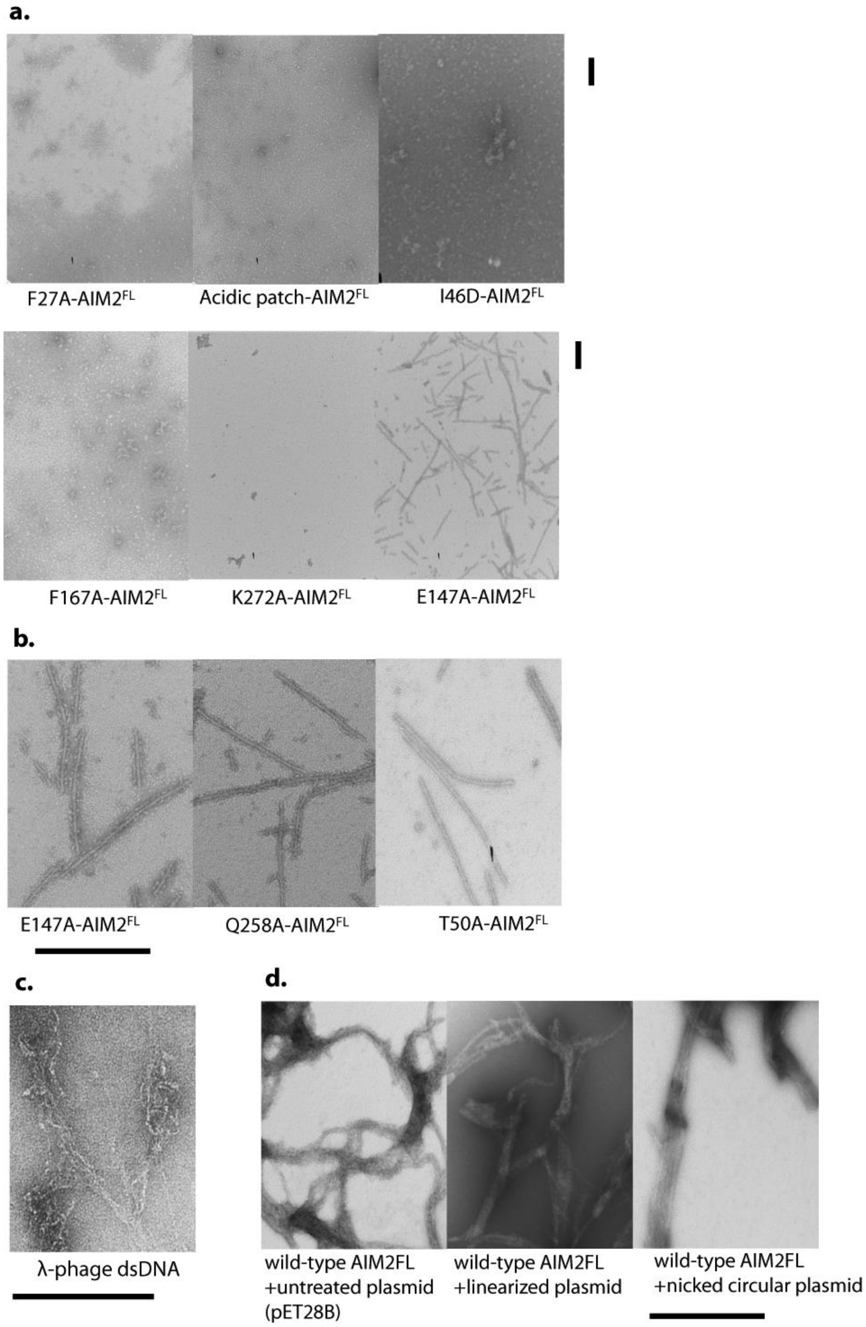

Supplementary Fig. 3. EM studies of AIM2 variants

(**a**) Electron micrographs of dsDNA-free AIM2^{FL} variants (2 µM). (**b**) Close-up views of dsDNA-free AIM2^{FL} mutant filaments. (**c**) λ-phage dsDNA. (**d**) Wild-type AIM2^{FL} filaments assembled on the indicated dsDNA. All scale bars indicate 100 nm.

Supplementary Table 1. AIM2 binding to FAM-dsDNA72 (160 mM KCl). ± indicates standard deviation. n ≥ 3.

Variant	K_D (nM)	Hill Constant
MBP-AIM2^{FL}	234 ± 42	1.4 ± 0.1
MBP-AIM2^{Hin}	584 ± 22	1.1 ± 0.2
AIM2^{FL}	≤ 3	NA
AIM2^{Hin}	212 ± 28	1.7 ± 0.2
L10-11A-AIM2^{FL}	52 ± 11	1.7 ± 0.2
Acidic Patch to Ala	37 ± 12	1.4 ± 0.2
F27A-AIM2^{FL}	> 200	NA
T50A-AIM2^{FL}	28 ± 7	NA
K173A-AIM2^{FL}	113 ± 18	1.5 ± 0.3
Q258A-AIM2^{FL}	37 ± 6	ND
K272A-AIM2^{FL}	53± 13	1.7 ± 0.4
E147A-AIM2^{Hin}	≥ 1500	ND
F167A-AIM2^{Hin}	≥ 2000	ND
K173A-AIM2^{Hin}	≥ 2500	ND
K272A-AIM2^{Hin}	≥ 2500	ND

Supplementary Table 2. AIM2 binding to FAM-dsDNA72 (400 mM KCl). ± indicates standard deviation. n ≥ 3.

Variant	K_D (nM)	Hill Constant
AIM2^{FL}	39 ± 12	1.5 ± 0.3
AIM2^{Hin}	N/B	ND
MBP-AIM2^{FL}	N/B	ND
MBP-AIM2^{Hin}	N/B	ND
L10-11A-AIM2^{FL}	N/B	ND
Acidic Patch to Ala	N/B	ND
F27A-AIM2^{FL}	N/B	ND
I46D-AIM2^{FL}	N/B	ND
T50A-AIM2^{FL}	95 ± 31	1.4 ± 0.1
E147A-AIM2^{FL}	143 ± 45	1.4 ± 0.2
F167A-AIM2^{FL}	N/B	ND
K173A-AIM2^{FL}	N/B	ND
Q258A-AIM2^{FL}	31 ± 5	1.4 ± 0.2

Supplementary Table 3. Binding of AIM2^{FL} toward various dsDNA lengths (400mM KCl).). ± indicates standard deviation. n ≥ 3.

DNA Length (bp)	K_D (nM)	Hill Constant
24	≥ 2000	N.D.
39	531 ± 93	1.2 ± 0.1
52	350 ± 12	1.1 ± 0.2
72	39 ± 12	1.5 ± 0.3

Supplementary Table 4. Binding of AIM2^{Hin} toward various dsDNA lengths (160mM KCl).). ± indicates standard deviation. n ≥ 3.

DNA Length (bp)	K_D (nM)	Hill Constant
10	>> 2000	
24	2110 ± 486	2.1 ± 0.4
39	1205 ± 339	2.2 ± 0.5
52	922 ± 168	1.4 ± 0.2
72	212 ± 33	2.1 ± 0.4

Supplementary Table 5. AIM2^{FL} Competition binding (160mM KCl).). ± indicates standard deviation. n ≥ 3.

DNA Length (bp)	IC_{50} (μg/mL)	Hill Constant
15	1695 ± 367	0.74 ± 0.17
24	1608 ± 151	0.69 ± 0.26
52	16 ± 6	0.97 ± 0.04
60	6 ± 2	1.00 ± 0.10
72	6.1 ± 1.8	1.17 ± 0.12
100	3.1 ± 0.3	0.8 ± 0.3
150	1.2 ± 0.3	1.5 ± 0.4
300	0.39 ± 0.24	2.24 ± 0.16

Supplementary Table 6. AIM2^{Hin} Competition binding (160mM KCl).). ± indicates standard deviation. n ≥ 3.

DNA Length (bp)	IC_{50} (µg/mL)	Hill Constant
15	805 ± 118	1.3 ± 0.1
24	365 ± 87	0.8 ± 0.3
39	119 ± 32	0.7 ± 0.2
52	31 ± 5	0.6 ± 0.2
60	14 ± 1	0.9 ± 0.4
72	25 ± 1	0.7 ± 0.1
100	9.1 ± 0.7	1.2 ± 0.7
150	4.1 ± 1.8	1.3 ± 0.3
300	4.8 ± 0.5	1.5 ± 0.3
600	5.3 ± 0.2	1.3 ± 0.1

Supplementary Table 7. AIM2^{FL} Competition binding (400mM KCl).). ± indicates standard deviation. n ≥ 3.

DNA Length (bp)	IC_{50} (µg/mL)	Hill Constant
15	328 ± 39	0.9 ± 0.4
24	237 ± 67	0.8 ± 0.4
39	16 ± 4	0.7 ± 0.3
52	5.2 ± 0.5	0.8 ± 0.2
60	2.6 ± 0.3	0.8 ± 0.1
72	0.5 ± 0.1	0.8 ± 0.4
100	0.29 ± 0.04	1.2 ± 0.1
150	0.14 ± 0.01	1.5 ± 0.1
300	0.09 ± 0.02	1.6 ± 0.1
600	0.09 ± 0.02	1.4 ± 0.3

Supplementary Table 8. AIM2^{FL} FRET Data (160mM KCl).). ± indicates standard deviation. n ≥ 3.

DNA Length (bp)	K_D (µg/mL)	Hill Constant
24	1.75 ± 0.57	0.9 ± 0.1
39	0.16 ± 0.01	1.4 ± 0.4
52	0.32 ± 0.01	1.9 ± 0.3
60	0.23 ± 0.06	1.6 ± 0.3
72	0.18 ± 0.02	2.1 ± 0.4
100	0.12 ± 0.02	1.6 ± 0.1
150	0.08 ± 0.01	1.5 ± 0.2
200	0.07 ± 0.02	1.5 ± 0.2
300	0.07 ± 0.02	1.2 ± 0.2
600	0.07 ± 0.01	2.6 ± 0.7
